# Swimming classes as campus blue space: a qualitative study of emotion regulation, social support and psychological resilience among university students

**DOI:** 10.3389/fpsyg.2026.1880166

**Published:** 2026-07-31

**Authors:** Yixu Wang, Shunli Lan

**Affiliations:** 1 College of Sports and Great Health, Sichuan Technology and Business University, Meishan, China; 2 Specialty Physical Education and Sports, Belarusian National Technical University, Minsk, Belarus

**Keywords:** blue space, emotion regulation, psychological resilience, social support, swimming

## Abstract

This qualitative study explores how university swimming classes are perceived by students as a campus blue-space setting for stress adjustment, emotion regulation, social support, and resilience-oriented coping. Semi-structured interviews were conducted with 24 first- and second-year undergraduates enrolled in compulsory physical education swimming classes at one private and one public university in Southwest China. Thematic analysis generated five contextually linked themes: academic and campus-life stress created demand for low-stigma recovery; water immersion, buoyancy, muffled sound, blue visual field, and rhythmic breathing were perceived to redirect attention from study-related rumination; peer encouragement and teacher reassurance fostered a socially safe learning climate; mastering fear, breathing, and stroke coordination was described as a resource for coping with academic and interpersonal challenges; and crowding, body exposure, assessment pressure, and facility conditions constrained restorative experiences. Rather than establishing causal effects, the study identifies participants’ perceived pathways and boundary conditions. The findings position campus swimming as a hybrid blue-space practice in which water-based affordances, physical activity, and relational pedagogy intersect. Implications are discussed cautiously for university physical education, student mental health promotion, and inclusive blue-space design.

## 1 Introduction

University students increasingly encounter dense academic workloads, competitive assessments, uncertain career expectations, and the social adjustments associated with leaving home. These pressures are not merely transient discomforts: international epidemiological evidence shows that the college years coincide with elevated onset and persistence of common mental disorders (Auerbach et al., 2016), while stressors related to academic performance and interpersonal transitions are consistently associated with poorer psychological functioning (Karyotaki et al., 2020). Recent work has further highlighted how academic stress is linked to diminished wellbeing among college students (Barbayannis et al., 2022). In China, the psychological vulnerability of university students has also been made visible through studies of anxiety, concentration difficulties, and disrupted routines during public health crises (Cao et al., 2020). These findings point to a practical challenge for higher education: how can campuses provide everyday, low-stigma environments through which students can recover from stress before distress becomes clinical?

Physical education (PE) is a promising but under-theorized answer to this question. Unlike counseling services, PE reaches large cohorts through a normal curricular channel and does not require students to identify themselves as having psychological problems. Systematic reviews show that physical activity interventions can improve mental health outcomes among undergraduate and higher education students, although the quality of implementation and theoretical specification remains uneven (Donnelly et al., 2024; Huang et al., 2024). Recent evidence from Chinese universities also suggests that PE may influence students’ psychological health through exercise behavior and social support (Han et al., 2025). Yet much of this literature treats PE as a general exposure measured by frequency, intensity, or satisfaction. Less is known about how a specific PE environment becomes meaningful to students as a site of emotion regulation, peer connection, and psychological restoration.

However, two analytic cautions are important. First, swimming classes combine multiple potential resources: general physical exercise, water-specific sensory affordances, skill learning, and social pedagogy. Without comparison groups, the unique effect of water cannot be isolated; it can only be examined through students’ own attributions. Second, qualitative interviews can identify perceived meanings and plausible pathways, but they cannot verify causal mechanisms or clinical effects. The present study therefore uses cautious language (e.g., “perceived,” “reported,” and “described”) and treats swimming as a context-dependent experience rather than a uniform intervention.

The present study focuses on swimming classes because they combine physical activity with a distinctive blue-space environment. Blue space refers to natural or human-made water environments that may support health through sensory exposure, movement, restoration, and social interaction. Cross-national and review evidence indicates associations between green and blue space exposure and mental health, while mechanistic reviews identify restoration, physical activity, environmental quality, and social contact as plausible pathways (Georgiou et al., 2021; White et al., 2021). On university campuses, however, the evidence base remains dominated by green landscapes, and recent reviews specifically identify campus blue spaces as an underdeveloped area (Aghabozorgi et al., 2024; Ribeiro et al., 2024). A campus swimming pool is therefore theoretically important: it is not simply a sports facility, but a structured, embodied, and socially organized water setting embedded in students’ weekly academic life.

Swimming may also offer psychological affordances that are difficult to capture in generic exercise measures. The water environment changes breathing, rhythm, posture, temperature, and bodily attention; it can produce sensations of buoyancy and containment while also requiring students to confront fear, embarrassment, or skill uncertainty. Evidence from blue-care interventions and open-water swimming suggests that water-based activities may support mood, wellbeing, and mental distress reduction, although much of this literature concerns outdoor or therapeutic contexts rather than compulsory campus PE (Britton et al., 2020; Overbury et al., 2023). Experimental research on repeated blue-space exposure also indicates potential mental health benefits when movement is situated near water (Vert et al., 2020), while earlier reviews established blue space as a meaningful, if still methodologically uneven, health resource (Gascon et al., 2017).

This study therefore asks under what conditions swimming classes are perceived to become a campus blue space for stress adjustment among Chinese undergraduates. Drawing on interviews with 24 first- and second-year students from a private university and a public university in Southwest China, it examines how students describe the emotional, social, and restorative meanings of swimming lessons while also identifying constraints that limit these meanings. Specifically, the study addresses three analytical research questions: (1) How do students describe perceived emotional shifts in swimming classes, and which elements do they attribute to water-based affordances rather than to exercise alone? (2) How are peer support, teacher behavior, and skill-learning routines connected with feelings of safety, shame reduction, and resilience-oriented coping? (3) What boundary conditions constrain the restorative potential and transferability of campus swimming? By shifting attention from whether PE is beneficial to how, for whom, and under what conditions swimming is experienced as a restorative blue-space practice, the study contributes to campus mental health, PE pedagogy, and environmental psychology.

## 2 Literature review

### 2.1 Blue space, restoration, and embodied movement

The conceptual foundation for viewing swimming classes as restorative campus blue space draws on two classic theories whose value lies in explaining why environments can shape psychological functioning. Stress recovery theory argues that non-threatening natural environments may elicit affective and physiological recovery from stress, whereas attention restoration theory proposes that certain environments renew directed attention by offering fascination and psychological distance from cognitive demands (Kaplan, 1995; Ulrich et al., 1991). These theories were developed before the current blue-space literature, but they remain valuable because they explain why a student might experience a swimming class as more than a physical task: water, sensory rhythm, and temporary separation from academic spaces can create a restorative break in attention and affect.

Recent blue-space research extends these classic ideas by identifying specific pathways through which water environments may support health. Reviews indicate that blue spaces are associated with psychological wellbeing and may operate through restoration, physical activity, social interaction, and environmental qualities such as sound, view, temperature, and perceived safety (Georgiou et al., 2021; White et al., 2021). Blue-care interventions further suggest that structured contact with water can promote psychosocial wellbeing, particularly when activity, place, and supportive relationships are combined (Britton et al., 2020). Although outdoor blue spaces are most studied, findings from repeated short walks near water show that even brief, recurrent blue-space exposure can improve subjective wellbeing and mood (Vert et al., 2020). For campus swimming, these findings suggest that the psychological effect of PE may not come only from energy expenditure but from the conjunction of movement and water-based environmental affordances.

At the same time, the literature does not support an unqualified claim that all water settings are restorative or that water effects can be separated easily from movement, novelty, or social context. Many studies are cross-sectional or conducted in outdoor leisure settings, and indoor swimming pools add institutional rules, chlorination, assessment, and body visibility. In this study, the blue-space lens is therefore used as an analytical framework for interpreting student accounts, not as evidence that water exposure itself caused mental-health change.

Swimming is also an embodied form of blue-space contact. Open-water swimming research reports improvements in mood and wellbeing and rich qualitative accounts of immersion, challenge, and connection, but it also emphasizes that evidence remains heterogeneous and often context-specific (Overbury et al., 2023). A university swimming class differs from voluntary open-water swimming in at least three ways: participation is curricular rather than leisure-based, the water space is institutional rather than natural, and peer comparison is built into the learning environment. These differences make campus swimming pedagogically distinctive. Students do not merely encounter water; they learn how to breathe, float, coordinate movement, and manage visibility before classmates. Thus, campus swimming can be conceptualized as a hybrid blue space in which environmental exposure, skill acquisition, and social evaluation occur simultaneously.

For the present analysis, this distinction required separating three overlapping components: (a) generic exercise features such as bodily movement, repetition, and post-exertion relaxation; (b) water-specific features such as immersion, buoyancy, muffled sound, temperature, and the blue visual field; and (c) class-based features such as teacher feedback, peer comparison, and formal assessment. This coding distinction directly addressed whether students themselves perceived swimming as different from land-based PE.

### 2.2 Emotion regulation in swimming classes

Emotion regulation refers to processes by which individuals monitor and modify emotional experiences, expressions, and physiological responses (McRae and Gross, 2020). Gross’s process model remains a classic framework because it distinguishes regulation strategies across stages such as situation selection, attentional deployment, cognitive reappraisal, and response modulation (Gross, 1998, 2015). In a swimming class, these processes may appear as students choosing to focus on breathing rather than examinations, reframing fear of water as manageable skill learning, or using bodily fatigue and rhythmic movement to down-regulate tension. The pool may therefore act as a situational scaffold for regulation: it narrows attention to immediate bodily coordination while providing visible markers of progress.

Because the present design did not measure physiological arousal or pre-post mood change, emotion regulation is treated as a reported process rather than an observed effect. Interview analysis therefore focused on how students narrated attention shift, breathing control, emotional calming, and reappraisal, while avoiding claims that these processes were objectively caused by swimming.

Emotion regulation is also closely related to resilience. Reviews of adversity and resilience suggest that the capacity to regulate emotionality helps individuals adapt to stress rather than become overwhelmed by it (Polizzi and Lynn, 2021). Swimming may support such adaptation because it exposes students to manageable difficulty. A novice swimmer may experience anxiety when floating, embarrassment when failing a stroke, or relief after completing a lap. When these emotions are processed in a safe instructional setting, they may become opportunities to practice persistence, self-efficacy, and flexible appraisal. This perspective shifts PE psychology from a simple exercise-improves-mood model toward a process account: swimming classes may build resilience by repeatedly linking emotional arousal with successful coping.

### 2.3 Social support and resilience in PE contexts

Social support theory further helps explain why the classroom form of swimming matters. Cohen and Wills’s (1985) buffering hypothesis is a classic account of how supportive relationships protect individuals from the harmful effects of stress, while later relational theories emphasize that support also enables thriving by helping people pursue challenges, exploration, and growth (Feeney and Collins, 2015). Recent reviews confirm that social support is strongly connected with college students’ mental health and wellbeing, including among students experiencing psychological difficulties (Li et al., 2025; Vicary et al., 2025). In swimming classes, support may appear through peer encouragement, shared laughter about mistakes, informal tutoring, partner safety checks, or teacher reassurance. These interactions can reduce the shame associated with low skill and transform the pool into a relational rather than merely individual space.

Resilience research complements this view by defining resilience not as a fixed trait but as adaptive functioning within systems of risk and support. Theoretical syntheses emphasize that resilience involves dynamic processes across personal, relational, and environmental systems, while sport and performance psychology highlights persistence, positive adaptation, and resource use under pressure (Fletcher and Sarkar, 2013; Masten, 2019; Southwick et al., 2014). In campus swimming, resilience may be visible when students reinterpret failure, continue attending despite fear, or transfer confidence from mastering a stroke to managing academic pressure. Importantly, resilience here is not assumed to be produced automatically by participation. It depends on whether the swimming environment is perceived as safe, inclusive, and pedagogically supportive.

Taken together, the literature suggests a promising but insufficiently examined link among blue space, PE, emotion regulation, social support, and resilience. Reviews of campus environments identify a need to examine blue spaces within university life rather than treating campus nature as generic greenness (Aghabozorgi et al., 2024; Ribeiro et al., 2024). PE intervention reviews call for stronger attention to mechanisms, context, and implementation, particularly because student mental health outcomes are shaped by more than activity dosage (Donnelly et al., 2024; Huang et al., 2024). Existing Chinese PE evidence indicates that social support and exercise behavior may connect PE with psychological health (Han et al., 2025), but students’ lived accounts of how this occurs in swimming classes remain limited. A qualitative interview design is therefore appropriate for identifying perceived experiential pathways and boundary conditions through which campus swimming may be interpreted as a restorative blue-space practice.

### 2.4 Conceptual framework

**FIGURE 1:**
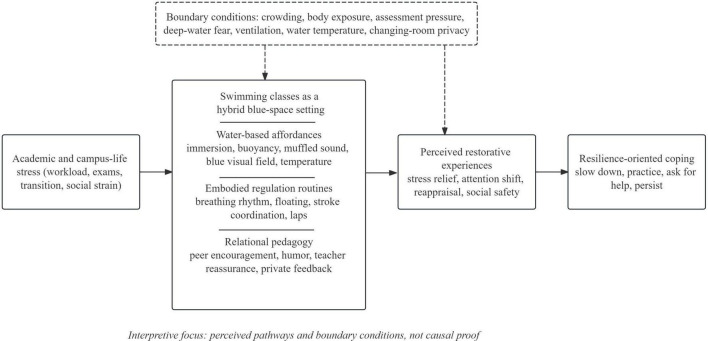
Conceptual framework of campus swimming classes as a hybrid blue-space practice.

Figure 1 synthesizes the sensitizing framework guiding the analysis. Academic and campus-life stress form the initial context; swimming classes are conceptualized as a hybrid blue-space setting in which water-based affordances, embodied regulation routines, and relational pedagogy interact. These processes may be associated with perceived stress relief, emotional reappraisal, social safety, and resilience-oriented coping, while crowding, body exposure, assessment pressure, deep-water fear, and facility conditions act as boundary conditions. The arrows in the figure represent interpretive relationships reported by participants rather than tested causal pathways.

## 3 Materials and methods

### 3.1 Research design and context

This study adopted a qualitative interview design to understand how undergraduate students interpret swimming classes as a setting for stress adjustment, emotion regulation, social support, and psychological resilience. A qualitative approach was appropriate because the study aimed to examine meanings, perceived mechanisms, and situated experiences rather than to test the statistical effect of swimming participation (Creswell and Poth, 2018). The design was interpretive and explanatory in a qualitative sense: it sought to build a conceptual account of reported pathways and boundary conditions rather than to estimate intervention effectiveness. The reporting and design logic were informed by the Standards for Reporting Qualitative Research and the Consolidated Criteria for Reporting Qualitative Research, which emphasize transparent sampling, interview procedures, analytic decisions, and reflexivity (O’Brien et al., 2014; Tong et al., 2007). The study was conducted in two universities in Southwest China: a private university (University A) and a public university (University B). In both institutions, swimming was offered as a compulsory component of the university-wide physical education curriculum for first- and second-year students; therefore, students beyond the second year were not included.

### 3.2 Participants and sampling

Participants were recruited through purposive criterion sampling. Eligibility required current enrollment in a university swimming class, first- or second-year undergraduate status, willingness to discuss emotional and social experiences of the class, and provision of informed consent. Recruitment information was distributed through class announcements and student networks, but participation was arranged directly with the research team so that teachers did not know which students volunteered. Students were told that participation or non-participation would not influence course grades, attendance records, or teacher evaluations. The final sample comprised 24 students. Eleven students came from University A, including five first-year and six second-year students; thirteen students came from University B, including seven first-year and six second-year students. Based on the interview records, the sample included 11 men and 13 women. Supplementary Appendix C presents the anonymized participant key used for quotations. In qualitative research, sample adequacy is judged by information power, analytic richness, and saturation rather than by statistical representativeness; empirical reviews suggest that many interview studies reach meaningful thematic saturation within sample sizes comparable to the present design (Hennink and Kaiser, 2022). Sampling and analysis were conducted iteratively. After 20 interviews, the research team observed that new transcripts were mainly repeating or elaborating existing codes across stress context, water affordances, support, mastery, and barriers. Four additional interviews were then coded to check whether experiences related to introversion, privacy, and sleep created new categories; they enriched the same framework without generating an additional theme. The team therefore judged thematic saturation adequate for the bounded aim of this study, while recognizing that the sample is not statistically representative. Table 1 summarizes the participants’ distribution by institution, institutional type, year level, and gender.

**TABLE 1:** Participant composition by institution and year level.

Institution	Institutional type	First-year students	Second-year students	Total	Male	Female
University A	Private university	5	6	11	5	6
University B	Public university	7	6	13	6	7
Total	–	12	12	24	11	13

### 3.3 Data collection

Semi-structured interviews were used because they allowed the interviewer to follow a common guide while giving students space to narrate personal experiences. Supplementary Appendix A presents the interview guide. The guide covered academic pressure and pre-class emotion, embodied experiences in water, emotion regulation during and after swimming, peer and teacher support, resilience transfer, and barriers to restoration. The guide was refined through research-team discussion to reduce leading questions and to make probes about stress, embarrassment, safety, and peer interaction easier to understand. During interviews, follow-up probes were used to clarify meanings when students described relaxation, anxiety, embarrassment, or coping transfer, so that the analysis would not infer mechanisms from vague positive statements alone. Interviews were conducted individually in a quiet setting selected to protect privacy. Before each interview, participants were informed of the study purpose, voluntary participation, confidentiality, the right to withdraw, and the anonymization of all identifying information. With permission, interviews were audio-recorded and later transcribed verbatim in Chinese. Field notes were written after each interview to capture contextual details, emotional tone, and preliminary analytic observations. For English-language reporting, illustrative quotations were translated after theme development and checked by a second researcher for semantic accuracy.

### 3.4 Data analysis

Data were analyzed through a theoretically informed thematic analysis. Braun and Clarke’s thematic approach was selected because it supports systematic coding while allowing themes to be developed through recursive movement between data, codes, and conceptual interpretation (Braun and Clarke, 2006, 2021). Two researchers processed the interview texts and used NVivo 14 for data management, coding, memo writing, and retrieval. Analysis began with repeated reading of all transcripts to identify initial impressions. The team initially developed a preliminary codebook based on theory, then iteratively revised it during coding to produce the final framework shown in Supplementary Appendix B. The framework functioned as a sensitizing structure rather than a closed taxonomy: deductive codes connected the data with established theory, while inductive coding preserved students’ own descriptions of the pool, the body, classmates, and recovery.

To make the coding process transparent, a meaning-bearing segment rather than a whole interview answer was used as the main coding unit. The preliminary codebook was organized around blue-space affordances, emotion regulation, social support, resilience, perceived outcomes, and barriers; after open coding, it was revised into the eight-category framework in Supplementary Appendix B. The two coders independently coded six transcripts selected to vary by university, year level, gender, and prior swimming experience. They then compared coded excerpts, clarified code boundaries, merged overlapping codes, and added decision rules. For example, a segment about water sound reducing rumination was double-coded only when it contained both an environmental description and an explicit regulation process; a segment about feeling relaxed after class was coded as an outcome unless it identified a mechanism. The remaining transcripts were coded after consensus was reached, and all disagreements were resolved through discussion and memo review rather than simple numerical reliability because the purpose was interpretive consistency and analytic depth.

During theme development, codes were compared within and across institution, year level, gender, and swimming background. Matrices were used to distinguish context (academic stress), affordance (water/sensory qualities), process (breathing, attention, reappraisal), relational condition (peer/teacher support), perceived outcome (relaxation/coping), and boundary condition (crowding, exposure, assessment, facilities). This context-process-outcome distinction reduced overlap among themes and helped prevent unsupported causal interpretation. Candidate themes were retained only when they were grounded in multiple transcripts or when a deviant/negative case clarified a boundary condition. Quotations in the Findings are attributed by participant codes in Supplementary Appendix C.

### 3.5 Trustworthiness and ethics

Trustworthiness was strengthened through credibility, dependability, confirmability, reflexivity, and transferability strategies derived from qualitative inquiry (Lincoln and Guba, 1985). Credibility was supported by prolonged engagement with transcripts, comparison of cases from two universities, attention to deviant and ambivalent cases, transparent quotation of participants’ accounts, and iterative checking of whether each theme was supported by multiple transcripts rather than by isolated positive examples. Dependability was supported by an audit trail containing the interview guide, coding framework, NVivo project records, codebook revisions, coding decision rules, and analytic memos.

Member checking was conducted after preliminary theme development. To protect anonymity and reduce participant burden, participants were not asked to approve full transcripts line by line. Instead, the research team shared concise Chinese summaries of the key interpretations related to participants’ accounts and the meanings of selected quotations, inviting participants to confirm whether these interpretations accurately represented their experiences and to correct any misunderstanding. No participant reported substantive disagreement with the summarized interpretations; minor wording clarifications were incorporated where appropriate. This summary-level member checking was combined with clarification probes during interviews, second-researcher checks of translated quotations, and conservative wording that reports experiences as perceived or described rather than as objectively verified outcomes.

Researcher reflexivity was addressed through memo writing and team discussion. One researcher had a sports and health background and therefore could have been inclined to interpret PE positively; the other researcher was outside the immediate PE teaching context and repeatedly questioned overly beneficial readings. Reflexive memos specifically documented assumptions about swimming, student mental health, body exposure, assessment pressure, and the risk of treating interview narratives as causal proof. Confirmability was strengthened by linking thematic claims to excerpts and by retaining negative cases such as crowding, body anxiety, deep-water fear, and assessment pressure. Transferability was supported by thick description of the institutional context, participant composition, curriculum constraints, and boundary conditions, while acknowledging that the two-university Southwest China sample limits generalization.

Ethical procedures included informed consent, voluntary participation, anonymization of institutions and participants, secure storage of recordings and transcripts, and removal of identifying details from quoted material. Because the topic involved stress and emotion, interviewers avoided diagnostic language and reminded participants that they could skip any question. Students who reported serious distress would be referred to university counseling or student-affairs support according to institutional procedures. The study was reviewed and approved by the Ethics Committee of College of Sports and Great Health, Sichuan Technology and Business University. All participants provided informed consent before the interviews.

## 4 Findings

Analysis generated five interrelated but analytically separated themes. Theme 1 describes the stress context that made a low-stigma recovery space meaningful; Theme 2 describes water-specific sensory affordances; Theme 3 describes reported attention and emotion-regulation processes; Theme 4 describes relational support and resilience-oriented coping; and Theme 5 describes boundary conditions that limited restoration. Across institutions, students described swimming classes as a weekly interruption to academic pressure, yet the restorative effect was not automatic. It depended on water-based sensory affordances, the regulation of attention and breathing, relational safety in the class, and the experience of mastering manageable difficulties. Differences were most visible by year level and prior swimming experience: first-year and zero-foundation students emphasized adaptation, homesickness, fear of water, and embarrassment, whereas second-year students more often connected swimming to future uncertainty, examination preparation, internships, competitions, or employment concerns. The themes below use translated participant quotations. Short quotations are bolded, and longer accounts are formatted as indented quotations with participant identifiers.

### 4.1 Theme 1: academic stress made students seek a low-stigma recovery space

Students rarely entered the swimming class from a neutral emotional state. Before class, they described academic overload, examinations, homework, laboratory reports, group work, career uncertainty, social adaptation, and sleep disturbance. First-year students often framed stress as transition from home or high school to university life. P15, a first-year woman from University B, reported homesickness, difficult professional courses, and insomnia; P14 described being unable to keep up with mathematics and computing assignments. Second-year students described heavier professional demands and future-oriented uncertainty. P11 linked poor interview performance with self-doubt about employment, while P20 associated failed competition plans with frustration and self-questioning. Swimming therefore became meaningful in participants’ narratives because it was inserted into an already stressed academic week, not because students were simply seeking recreation.

Several participants called the class “the most relaxing time each week” or “a healing time.” These phrases were not casual compliments; they signaled that students valued the pool as an ordinary, curriculum-based pause from pressure. P09 described the class as an “emotional shelter” because being in the water allowed her to stop ruminating on roommate conflict and exam preparation. P10 called it a “stress-relief device” that cleared anxiety about postgraduate entrance plans. Such language suggested a low-stigma quality: students did not need to identify themselves as psychologically distressed in order to access the pool. They could attend because it was a PE class, while privately using it to recover.

*Before swimming class, I had usually just finished reading postgraduate entrance materials or writing assignments. My head was heavy, my heart felt panicked, and my whole body was tight. Once I entered the water, I did not think about the future for a while. I just focused on the movement, and after a few laps the panic settled down. Then I could sort out my direction more rationally*. (P10, University A, male, second year)

The data also showed that stress was both academic and social. Several first-year participants associated distress with leaving home, difficulty fitting into dormitory life, or not knowing how to ask questions. P24, who described herself as sensitive and slow to integrate into groups, said that before class she often felt empty, tired, and irritable. For her, the swimming class was valuable because it did not demand immediate verbal confidence. This pattern indicates that campus PE may support mental health not only by increasing physical activity but also by offering socially acceptable breaks from academic and interpersonal vigilance.

### 4.2 Theme 2: water immersion was perceived to shift attention from rumination to the present body

Participants repeatedly described the pool through sensory contrasts with classrooms, dormitories, libraries, and laboratories. The most common contrast was sound: students heard water rather than classroom noise, phone notifications, or group discussion. They also mentioned temperature, buoyancy, blue visual space, the feel of water around the body, and the requirement to coordinate breathing with movement. These affordances redirected attention from abstract worries to concrete bodily tasks. P03 said that in the pool, “study pressure could not be heard or remembered.” P02 similarly felt that the blue water placed unfinished tasks “outside the pool” and drew attention to her own movements. Although physical activity in general can support mood, participants’ accounts repeatedly identified water-specific cues rather than effort alone: muffled sound, body being wrapped, blue visual field, floating, coolness, and the need to breathe with water movement. These attributions help distinguish the perceived blue-space component from generic exercise, although they do not prove a separate water effect.

After I entered the water, I could clearly feel my body being wrapped by it. The touch was soft, and the sound insulation was obvious; I could not hear the noisy voices around me, so the world suddenly became quiet. When I practiced holding my breath and floating, I had to focus on my breathing and could not keep thinking randomly. My tense shoulders and stiff body slowly relaxed, and the loneliness in my heart also weakened. (P24, University B, female, first year)

Some students explicitly contrasted swimming with land-based PE. P03 and P06 described swimming as gentler, cooler, or less confrontational than running and ball games, while P23 valued swimming as a sport that did not require forced social interaction. These comparisons do not establish superiority over other activities, but they show that students themselves differentiated swimming through the water medium, quieter environment, and lower-contact format.

This theme also included ambivalence. Novices often reported initial fear, coldness, or bodily tension. P07 said that when she first entered the water, she was “stiff all over” and gripped the pool edge. P04 similarly felt afraid of deep water and worried about choking. Yet these negative sensations did not necessarily cancel restoration. When teachers slowed the pace and students experienced buoyancy safely, fear was gradually transformed into attention to breathing and floating. In this sense, the water environment did not simply soothe; it demanded bodily adaptation, and that adaptation became part of the restorative process.

### 4.3 Theme 3: rhythmic breathing and swimming routines were perceived to support emotion regulation

Students connected emotional change to breathing rhythm, repeated laps, and the temporary suspension of academic tasks. They did not portray swimming as instant happiness. Rather, they described a sequence: entering the water while tense, focusing on breathing and movement, reducing rumination, and returning to academic work with greater clarity. P01 said that after a poor mathematics quiz, several laps helped him stop “drilling into the dead end of failing the test.” P03 described returning to a difficult mathematics problem and suddenly finding a solution. P05 similarly reported that after code errors in a course design project, steady swimming helped him identify the problem more efficiently than continuing to force himself at the desk.

*I had just finished a mathematics quiz and did very badly. I felt panicked and irritated, as if I could not keep up no matter how hard I studied. That day we had swimming class. After I entered the water, I slowly swam while following the rhythm of breathing in and out. After a few laps, the anxious feeling gradually faded. After getting out of the pool, my head was very clear, and I could calmly think about what I had not learned well. It was much more useful than sitting in the dormitory in a daze.* (P01, University A, male, first year)

The regulation described here should be understood as a participant-reported process. The data do not verify physiological calming; nevertheless, students consistently narrated attentional deployment, breathing rhythm, emotional settling, and cognitive reappraisal. They repeatedly said that they could not worry about examinations and coordinate breathing at the same time. The bodily demand of swimming therefore appeared in their accounts as an attentional boundary. Several students also described a post-class shift from helplessness to practical problem solving. P21, after dealing with a rejected paper draft and sudden club work, felt that one lap after another helped her calm irritation and arrange tasks again. P18, whose English mock examination result was disappointing, said that breathing with the stroke reduced self-denial and enabled a more rational revision plan. These accounts show why swimming felt different from passive distraction: the body had to participate in regulation through breathing, rhythm, and movement.

Emotion regulation also included students’ reappraisal of failure. Poor test performance, group-work conflict, unsuccessful competition plans, and internship rejection were often described as emotionally consuming before class. After swimming, participants did not claim that the external problems disappeared, but they were less likely to interpret them as proof of personal inadequacy. The pool created a short interval in which students could separate the stressful event from the self-judgment attached to it. P13 said that after a difficult group-work conflict and homesickness, quiet floating helped her stop ruminating and communicate with teammates more calmly. In this way, swimming was perceived as a curricular routine for emotional resetting rather than as a direct cure for stress.

### 4.4 Theme 4: peer support, teacher reassurance, and mastery were perceived as resilience resources

Social support was central to students’ willingness to stay in the water, especially for novices and students with body anxiety or fear of choking. Peers provided practical help by holding hands, explaining breathing skills, checking safety, correcting strokes, and sharing tips. They also provided emotional support through humor. Many participants stressed that classmates joked after mistakes but did not ridicule them. P02 said that choking became less embarrassing because classmates would laugh together and continue practicing; P14 emphasized that “no one laughed at beginners.” These interactions turned the visible vulnerability of learning to swim into a shared classroom experience rather than an individual failure.

*The girls in my class were very kind. I often choked because I was not good at breathing, and the girls next to me would pull me to practice and share small tips. When we made mistakes, we laughed with each other, so it did not feel embarrassing. The teacher was also gentle. She noticed that we were afraid of water and felt shy, taught the movements patiently, and encouraged us instead of criticizing us for learning slowly. In the water I felt very safe.* (P02, University A, female, first year)

Teacher behavior shaped whether swimming was interpreted as challenge or threat. Participants valued teachers who did not rush, shame, or overemphasize grading. P04 remembered the teacher saying “Do not be afraid; I am here” while staying with her in the shallow area. P03 appreciated that the teacher corrected errors without pressure and gave free-practice time. For introverted students, support also meant non-intrusive respect. P23 valued a classroom in which peers practiced separately and the teacher corrected mistakes quietly rather than publicly. The social climate therefore had more than a pleasant atmosphere; it was a condition of psychological safety.

Support and mastery were closely linked in participants’ accounts. Students repeatedly described moving from fear, awkwardness, or repeated failure toward small but visible progress: floating independently, mastering breathing, entering deeper water, coordinating a turn, or swimming several meters. These achievements were psychologically important because they converted anxiety into perceived evidence of competence. P01 concluded from learning to float and breathe that frightening things could be overcome slowly. P08, who repeatedly failed a turn-and-push movement before mastering it, said the process taught him that setbacks are normal and that difficult academic problems can be broken down and practiced. The class therefore did not merely relieve stress in the moment for these students; it offered a concrete model of persistence under manageable difficulty.

At the beginning I was afraid of water and did not dare to put my head under. I practiced many times and wanted to give up, but later I gradually learned to float and breathe. Suddenly I felt that things I was afraid of could be overcome slowly. Now when I meet a learning problem, such as not understanding mathematics, I do not panic or give up immediately. I think of learning swimming: take it step by step, practice more, ask more, and the mindset becomes steadier. (P01, University A, male, first year)

Resilience transfer was especially clear when students themselves connected swimming difficulties with academic and interpersonal coping. P22 learned from repeated floating practice that many things cannot be rushed; P24 connected overcoming fear of submerging her head with accepting her introverted personality and asking for help in study; P11 related repeated breathing practice to persistence after internship setbacks. Importantly, participants did not portray themselves as transformed into permanently resilient people. Instead, they described practical changes in appraisal: slow down, divide the problem, practice again, ask for help, and avoid self-denial. These micro-transfers suggest that resilience in PE can be understood as a perceived coping orientation rather than a fixed personality trait or an objectively measured outcome. It should be noted that not all students articulated a clear transfer; some described only a general sense of relaxation or patience. Nonetheless, among those who connected swimming to academic coping, the pattern was consistent and vivid.

### 4.5 Theme 5: crowding, body exposure, assessment, and facilities limited restoration

The same setting that enabled recovery could also produce new pressure. The most common barrier was crowding. Students from both universities said that crowded lanes restricted movement, increased fear of collision, and reduced the sense of quiet. For novice students, crowded shallow areas undermined safety; for more experienced swimmers, narrow lanes interrupted rhythm. P23 said that crowded lanes made him tense because he worried about hitting others. Several students recommended small classes, staggered scheduling, separate novice practice zones, or leisure lanes for slow swimming.

*The biggest problem is that there are too many people in class and the lanes are crowded. When I swim, I am afraid of bumping into others, so I become tense without noticing it, and that breaks the feeling of relaxation. The second issue is the final speed assessment. For someone like me who mainly wants to relax, it creates pressure. I hope there can be leisure lanes and assessment lanes, with a quiet space for students who just want to swim slowly.* (P23, University B, male, second year)

Body exposure was another boundary condition, especially among women. P02, P06, P11, P13, P15, P17, P19, and P21 described discomfort in swimwear or worry about being observed. P21 said that swimwear made her “not very comfortable” and increased psychological burden when combined with strict assessment. Assessment stress was reported by novices who feared failing and by experienced students who worried about speed or standardization. Chlorine odor, ventilation, unstable water temperature, and crowded changing rooms were also mentioned. These barriers show that blue-space exposure is not inherently restorative; it becomes restorative when institutional design protects safety, privacy, pacing, and dignity.

Negative cases sharpened the analysis rather than contradicting the overall pattern. Students who loved swimming still requested better ventilation or flexible assessment, while students who feared water could experience relief only when teachers stayed patient and expectations remained realistic. The findings therefore support a conditional interpretive model: swimming classes were perceived to function as campus blue space when environmental affordances, social support, and pedagogical design aligned. When they did not, the same pool could reproduce pressure through crowding, comparison, body surveillance, or performance anxiety.

## 5 Discussion

### 5.1 Campus swimming as a hybrid blue space

This study extends blue-space scholarship by showing how a university swimming pool can operate as a hybrid blue space: institutional rather than natural, curricular rather than voluntary, and pedagogically structured rather than purely recreational. Existing blue-space research emphasizes restoration, physical activity, social interaction, and environmental qualities such as sound, temperature, view, and perceived safety (Georgiou et al., 2021; White et al., 2021). The present findings are consistent with these proposed pathways but specify how they were experienced and narrated in campus PE. Students did not simply report that exercise improved mood; they explained how water sound, buoyancy, coolness, visual blue space, and bodily containment separated them from classrooms, dormitories, libraries, and laboratories. This contributes to campus-environment reviews that call for more attention to university blue spaces rather than an exclusive focus on green settings (Aghabozorgi et al., 2024; Ribeiro et al., 2024).

The findings also complicate the assumption that blue space is restorative because it is natural. The swimming pool was human-made, rule-bound, and sometimes crowded, yet participants experienced it as psychologically distinct from academic space. Its restorative quality was described as emerging from a combination of sensory affordances and institutional scheduling: the class appeared in the weekly timetable as a legitimate pause from study. This matters for universities because it suggests that mental health promotion may be embedded in ordinary curricular environments, but only when students experience those environments as safe, embodied, and socially acceptable.

### 5.2 Distinguishing water-specific affordances from exercise and pedagogy

This study cannot claim that water produced benefits above and beyond other exercise because no comparison group was included. What it can offer is an analytic differentiation within students’ narratives. Participants attributed some changes to exercise-like routines (movement, repetition, fatigue), some to water-specific affordances (immersion, buoyancy, muffled sound, blue visual field, temperature, and breathing in water), and some to pedagogical or social context (teacher reassurance, peer humor, flexible pacing, and assessment). The concept of a hybrid blue space captures this combination and avoids reducing swimming either to general exercise or to water exposure alone.

Practically, this means universities should not assume that any pool automatically supports wellbeing. The evidence suggests that the pool becomes restorative only when water affordances are protected by environmental quality and psychologically safe teaching. Conversely, crowding, chlorination, body exposure, and rigid speed assessment can turn the same setting into stress. Thus, the contribution lies in specifying the conditions under which campus swimming was perceived as supportive.

### 5.3 Emotion regulation through embodied routines

The study offers a process-based interpretation of how students perceived swimming to assist emotion regulation. Gross’s model highlights that people regulate emotion through attention, appraisal, and responses across different stages of emotional experience (Gross, 1998, 2015; McRae and Gross, 2020). Students’ accounts mapped onto these processes, but in a strongly embodied way. Attention was described as shifting because breathing, floating, and stroke coordination demanded immediate bodily awareness. Calming was narrated through repeated rhythm and the sensation of weight being reduced by buoyancy. Reappraisal appeared after class, when students returned to failed quizzes, papers, coding tasks, or interpersonal conflict with less self-blame and more practical planning. These are perceived processes rather than measured causal mechanisms.

This helps explain why students contrasted swimming with passive distraction, such as staying in the dormitory. In the pool, the body required active participation in regulation; students could not simply withdraw into rumination. The findings are consistent with resilience research showing that emotion regulation is central to adaptive coping under stress (Polizzi and Lynn, 2021), while adding that PE classes may scaffold reported regulation through repeated bodily routines. Campus swimming may be especially meaningful because breathing is not a background feature but a visible and teachable skill. When students learn to slow down and coordinate breathing under mild challenge, they also rehearse a coping sequence that they later described as transferable: notice tension, focus on rhythm, continue gradually, and return to the problem with a steadier appraisal.

### 5.4 Social support, pedagogical safety, and resilience

The results are consistent with the importance of social support in student mental health while specifying how support was embodied in PE. Cohen and Wills’s (1985) buffering hypothesis suggests that supportive relationships reduce the harmful effects of stress, and later thriving perspectives emphasize that support helps individuals pursue growth under challenge (Feeney and Collins, 2015). In the swimming classes, support appeared as peer tutoring, joking that reduced embarrassment, hands-on help in shallow water, teacher patience, private correction, and permission to progress slowly. These interactions reduced the shame attached to being unable to swim and changed the meaning of failure from personal inadequacy to shared learning.

The resilience finding should be interpreted carefully. Swimming did not automatically produce resilience, nor did students claim that one class solved academic stress. Instead, resilience-oriented coping was described as emerging from the interaction among manageable difficulty, visible progress, and social safety. This is consistent with developmental and sport psychology accounts that understand resilience as adaptive functioning within systems of support rather than as an isolated individual trait (Fletcher and Sarkar, 2013; Masten, 2019; Southwick et al., 2014). The pool gave students repeated experiences of being anxious, practicing, receiving support, improving slightly, and reinterpreting difficulty. Such small cycles may be pedagogically powerful because they are concrete, bodily felt, and potentially transferable to academic problems. Not all participants reported such direct transfer with equal clarity; therefore, the finding should be read as a perceived coping pathway, not as evidence of generalized resilience improvement.

### 5.5 Boundary conditions and equity-sensitive PE design

A major contribution of this study is that it avoids romanticizing swimming as universally restorative. Crowding, assessment pressure, body exposure, chlorine odor, changing-room privacy, and deep-water fear all limited the restorative effect. These boundary conditions are theoretically important because blue-space mechanisms depend on perceived safety and environmental quality (Georgiou et al., 2021; White et al., 2021). A crowded or surveillance-like pool can undermine fascination, psychological distance, and recovery. Similarly, a grading system that prioritizes speed may convert a recovery-oriented class into another performance pressure, especially for zero-foundation students.

For practice, universities should treat the following recommendations as evidence-informed design hypotheses rather than proven interventions. Each recommendation follows directly from a barrier or support condition reported by participants: smaller class sizes and staggered scheduling respond to crowding; stable water temperature and ventilation respond to sensory discomfort; marked novice and leisure lanes respond to fear of collision and differing skill levels; more private changing areas and gender-sensitive options respond to body exposure concerns; and assessment criteria that consider individual progress respond to performance pressure. Teachers also need training in psychological safety: slow pacing, supportive language, private feedback, and explicit normalization of choking, fear, and awkwardness can protect students’ dignity. These strategies are not separate from PE quality; they are conditions under which swimming was perceived as more likely to function as restorative campus blue space.

### 5.6 Limitations and future research

Several limitations should be acknowledged. The study was conducted in two universities in Southwest China, so findings may not transfer directly to institutions with different PE curricula, pool access, class sizes, cultural norms around body exposure, or student support systems. The sample was small and purposive; it provided information-rich accounts but not statistical representativeness. The design relied on retrospective interview accounts and therefore identified perceived mechanisms rather than measuring physiological stress recovery, clinical mental health outcomes, or pre-post mood change. Although summary-level member checking was conducted, it was limited to participants’ confirmation of key interpretations and selected quotation meanings rather than repeated longitudinal validation or independent verification of psychological outcomes. The study also lacked comparison groups such as running, ball games, land-based PE, or non-PE blue-space exposure; consequently, it cannot isolate the unique effect of water from general exercise, novelty, or supportive pedagogy. Future studies could combine interviews with experience sampling, mood scales, sleep indicators, or physiological measures before and after swimming. Longitudinal research could examine whether repeated swimming participation changes coping orientation over a semester and whether patterns differ by gender, prior swimming experience, disability, introversion, or assessment design. Comparative studies across swimming, running, ball games, and campus green-space activities would further clarify which mechanisms are specific to water-based PE and which reflect broader benefits of structured physical activity.

## 6 Conclusion

This qualitative study suggests that university swimming classes can be perceived as campus blue space when water-based sensory affordances, embodied regulation routines, social support, and pedagogical safety are brought together. For the 24 interviewed students, swimming was meaningful because it interrupted academic and interpersonal pressure through an ordinary curricular channel. The pool separated students from classrooms, assignments, dormitory tensions, and future uncertainty, while water sound, buoyancy, temperature, and rhythmic breathing redirected attention to the present body. These experiences were described as helping students calm anxiety, reduce rumination, and return to academic or social problems with clearer appraisal. Equally important, the restorative meaning was relational: peer encouragement, shared humor, teacher patience, and private feedback reduced embarrassment and enabled students to persist through fear, choking, or repeated failure. Mastery of floating, breathing, and stroke coordination then became, for some students, a small but concrete model of resilience-oriented coping. However, the findings also demonstrate that swimming is not automatically therapeutic. Crowded lanes, body exposure, strict skill assessment, deep-water fear, poor ventilation, and limited privacy can turn a potential recovery space into another source of pressure. The broader implication is that student mental health promotion can be embedded in everyday curricular spaces, but only when those spaces are designed with environmental quality, equity, privacy, and psychological safety in mind. Campus swimming should therefore be viewed not only as technical skill instruction but also as a pedagogical and environmental resource whose benefits are conditional, situated, and worthy of further comparative research.

## Data Availability

The original contributions presented in this study are included in the article/Supplementary material, further inquiries can be directed to the corresponding authors.
