# Supplementary material for: Swimming classes as campus blue space: a qualitative study of emotion regulation, social support and psychological resilience among university students
Source: Front Psychol. 2026 Jul 31;17:1880166. doi: 10.3389/fpsyg.2026.1880166 (PMC13473352; doi:10.3389/fpsyg.2026.1880166)
Supplement: Supplementary file 1 [file Supplementary_file_1.docx]

# Appendix A. Semi-Structured Interview Guide

The guide was used flexibly in individual interviews with students enrolled in university swimming classes. Probes were adapted according to each participant's response while maintaining coverage of the core domains.

| **Domain** | **Core interview questions** | **Suggested probes** |
| --- | --- | --- |
| Opening and rapport | Please briefly introduce your year level, swimming-learning background, and how you chose or entered this swimming class. | Prior swimming experience; expectations before the course; feelings about compulsory or elective PE. |
| Academic pressure and emotional baseline | What kinds of academic or campus-life pressures have you experienced recently? How did you usually feel before attending swimming class? | Workload, examinations, assignments, adaptation to university life, sleep, anxiety, irritability, fatigue. |
| Embodied blue-space experience | When you enter the pool or begin swimming, what bodily or sensory changes do you notice? | Water temperature, buoyancy, breathing, rhythm, sound, visual blue space, attention shift, feeling separated from study spaces. |
| Emotion regulation during and after class | Can you describe a specific moment when swimming helped you calm down, release pressure, or change your mood? | Attention, breathing, reappraisal, emotional release, post-class clarity, comparison with other PE classes. |
| Peer and teacher support | How do classmates or teachers influence your feelings and learning in swimming class? | Encouragement, joking, partner practice, safety reminders, embarrassment, comparison, belonging, teacher feedback. |
| Resilience and transfer | Has learning to swim or overcoming difficulties in the pool influenced how you deal with academic or life stress? | Fear of water, failure, persistence, confidence, coping transfer, self-efficacy, future participation. |
| Barriers and improvements | Are there any aspects of swimming class that create stress or reduce its restorative effect? What would make the class more supportive? | Facilities, timetable, safety, body image, gender comfort, teaching pace, assessment, privacy. |
| Closing | Is there anything else about swimming, stress relief, emotions, peer relationships, or psychological recovery that you would like to add? | Invite participant-led meanings; ask permission for follow-up clarification or member checking if needed. |

# Appendix B. Revised Coding Framework for NVivo 14

This revised framework was updated after coding the interview data. It combines deductive categories from blue-space theory, emotion regulation, social support, and resilience with inductive codes derived from participants' accounts. Coders applied the most specific subcode possible and wrote analytic memos when a segment bridged multiple categories.

| **Analytic category** | **Operational definition** | **Refined codes / indicators** | **Coding decision rules** | **Illustrative participants** |
| --- | --- | --- | --- | --- |
| Academic stress and pre-class affect | Segments describing stressors and emotional states before swimming. | Academic workload; quizzes/exams; assignments; experimental reports; group work; career uncertainty; homesickness; dormitory or peer conflict; fatigue; insomnia; irritability; anxiety. | Code when the segment establishes the stress context from which swimming-class recovery is interpreted. Do not code general course description unless emotional burden is stated. | P01, P04, P10, P15, P20, P24 |
| Embodied blue-space affordances | Segments describing water, pool space, sensory qualities, and the environmental difference between swimming and academic settings. | Water immersion; buoyancy; body being wrapped; blue visual field; water sound; temperature; quietness; separation from classrooms/dormitories; perceived safety; initial coldness or fear. | Code positive and negative blue-space meanings. If water is described as relaxing through attention or breathing, double-code with emotion regulation. | P02, P03, P07, P13, P19, P24 |
| Attention and emotion regulation processes | Segments showing how students manage, shift, express, or reinterpret emotions through swimming. | Attentional deployment; breathing rhythm; perceived bodily calming; stress detachment; reduction of rumination; post-class clarity; emotional release; reappraisal of failure; academic re-engagement. | Code only when a regulation process or mood change is described. Avoid assuming regulation from participation alone. | P01, P05, P08, P13, P18, P21 |
| Peer and teacher support | Segments describing peer, teacher, or group interactions that influence safety, learning, belonging, or shame. | Peer encouragement; hand-holding; sharing tips; joking/laughter; partner protection; teacher patience; private correction; reassurance; belonging; non-intrusive co-presence; comparison pressure. | Code supportive and unsupportive interactions. Distinguish emotional support from practical skill support when possible. | P02, P04, P12, P14, P21, P23 |
| Mastery, fear management, and resilience transfer | Segments linking skill acquisition, repeated practice, or overcoming fear with confidence and coping outside the pool. | Floating independently; breathing mastery; entering deep water; repeated practice; persistence after failure; self-efficacy; step-by-step coping; transfer to study, exams, group work, interviews, competitions, or interpersonal conflict. | Code transfer only when the participant explicitly connects swimming experience with broader coping or resilience. | P01, P08, P11, P18, P22, P24 |
| Perceived restoration and outcomes | Segments describing perceived psychological or practical outcomes after swimming. | Relaxation; improved mood; mental clarity; reduced self-denial; renewed energy; sleep improvement; willingness to continue swimming; emotional shelter; healing time; stress-relief routine. | Code as perceived outcomes, not causal proof. If specific mechanisms are present, also code with corresponding process category. | P03, P06, P09, P10, P13, P24 |
| Barriers and boundary conditions | Segments describing factors that create pressure or reduce restorative effects. | Crowding; narrow lanes; collision risk; body exposure; gender comfort; swimwear anxiety; assessment stress; speed standards; deep-water fear; chlorine odor; ventilation; water temperature; changing-room privacy. | Use to identify when swimming does not function as restorative blue space or requires design adaptation. | P02, P06, P07, P11, P17, P23 |
| Institutional and curriculum context | Segments referring to institutional type, compulsory PE, scheduling, assessment rules, class management, or pool resources. | Private/public university context; first-/second-year PE requirement; compulsory/elective route; random allocation; class size; attendance; grading; pool access. | Use for cross-case comparison and methodological description. Do not code routine institutional details unless related to experience. | P01, P05, P12, P16, P21, P23 |

# Appendix C. Participant Coding Key for Quotations

Participants are anonymized as P01-P24 for use in the Findings. For quotations, use the format P01 (University A, male, first year), with the detailed participant key below. The source IDs are internal transcript identifiers and can be removed if the journal requires stricter blinded review.

| **Participant code** | **Internal source ID** | **Institution** | **Institutional type** | **Gender** | **Year level** |
| --- | --- | --- | --- | --- | --- |
| P01 | A-S01 | University A | Private | Male | First year |
| P02 | A-S02 | University A | Private | Female | First year |
| P03 | A-S03 | University A | Private | Male | First year |
| P04 | A-S04 | University A | Private | Female | First year |
| P05 | A-S05 | University A | Private | Male | Second year |
| P06 | A-S06 | University A | Private | Female | Second year |
| P07 | A-S07 | University A | Private | Female | First year |
| P08 | A-S08 | University A | Private | Male | Second year |
| P09 | A-S09 | University A | Private | Female | Second year |
| P10 | A-S10 | University A | Private | Male | Second year |
| P11 | A-S11 | University A | Private | Female | Second year |
| P12 | B-S01 | University B | Public | Male | First year |
| P13 | B-S02 | University B | Public | Female | First year |
| P14 | B-S03 | University B | Public | Male | First year |
| P15 | B-S04 | University B | Public | Female | First year |
| P16 | B-S05 | University B | Public | Male | First year |
| P17 | B-S06 | University B | Public | Female | First year |
| P18 | B-S07 | University B | Public | Male | Second year |
| P19 | B-S08 | University B | Public | Female | Second year |
| P20 | B-S09 | University B | Public | Male | Second year |
| P21 | B-S10 | University B | Public | Female | Second year |
| P22 | B-S11 | University B | Public | Female | Second year |
| P23 | B-S12 | University B | Public | Male | Second year |
| P24 | B-S13 | University B | Public | Female | First year |
